# Metabolic engineering of tobacco for heterologous production of rare ginsenosides CK and Rh2

**DOI:** 10.3389/fpls.2026.1884201

**Published:** 2026-07-07

**Authors:** Linggai Cao, Xuemeng Li, Shanshan Lang, Yan Chen, Ping Dong, Jie Zhang, Jie Liu, Pan Zhang, Shizhou Yu, Jianfeng Zhang

**Affiliations:** 1 Key Laboratory of Biosynthesis and Biomanufacturing in Model Plants (Beijing Life Science Academy), Ministry of Industry and Information Technology, Beijing, China; 2 Key Laboratory of Tobacco Biological Breeding in Mountainous Ecological Regions, Guizhou Academy of Tobacco Science, Guiyang, China; 3 China Tobacco Guangdong Industrial Co., Ltd., Guangzhou, China; 4 China Tobacco Hubei Industrial Co., Ltd., Wuhan, China

**Keywords:** saponin, heterologous production, metabolic engineering, synthetic biology, tobacco

## Abstract

Rare ginsenosides, such as compound K (CK) and Rh2, possess potent pharmacological activities but are present at extremely low levels in natural *Panax ginseng*. Here, we report the heterologous biosynthesis of CK and Rh2 in tobacco (*Nicotiana benthamiana* and *N. tabacum* K326) by introducing three key genes—*DDS*, *CYP716A47*, and either *UGTPg1* (for CK) or *UGTPg45* (for Rh2)—under the control of the CaMV 35S promoter. Transgenic T2 lines were generated and characterized by PCR, RT-PCR, RT-qPCR, and LC–MS with full method validation. In *N. benthamiana*, CK accumulated preferentially in roots, reaching up to 47.87 μg/g dry weight (DW), whereas K326 showed higher Rh2 accumulation, up to 8.11 μg/g DW in roots. Root tissues consistently contained greater ginsenoside levels than leaves across both species. Topping (apical bud removal) led to increased CK and Rh2 levels in K326 lines, though the effect should be interpreted cautiously due to the lack of mock-wounding and time-course controls. Callus cultures derived from transgenic leaves enabled ginsenoside production (CK: 20.52 μg/g DW; Rh2: 0.98 μg/g DW), providing a proof-of-concept for *in vitro* production. These results demonstrate that tobacco can serve as an efficient plant chassis for the heterologous production of rare ginsenosides and highlight the importance of species-specific chassis selection, while also calling for additional studies to overcome current limitations in host comparison, topping validation, and callus scalability.

## 1 Introduction

Ginsenosides, the principal pharmacologically active constituents of *Panax ginseng*, exhibit diverse biological activities that underpin their broad therapeutic potential (Cao et al., 2020; Lee et al., 2025). Among them, rare ginsenosides such as compound K (CK) and Rh2 display enhanced bioactivity and improved bioavailability relative to their more abundant glycosylated precursors (Paek et al., 2006). However, their trace natural abundance, combined with the technical and economic challenges associated with chemical synthesis, severely restricts their large-scale utilization in the pharmaceutical and nutraceutical industries (Fan et al., 2024).

Metabolic engineering presents a sustainable and scalable alternative for producing these high-value compounds (Courdavault et al., 2021). Recent advances emphasize the use of plant cell, tissue, and hairy root cultures, engineered microbial hosts, and heterologous expression in plant chassis to circumvent conventional cultivation barriers (Xue et al., 2025). Although microbial systems have been exploited for ginsenoside biosynthesis, their performance is often constrained by inefficient expression of plant-derived cytochrome P450 enzymes, elevated fermentation costs, and contamination risks (Paramasivan and Mutturi, 2017; Han and Miao, 2024). In contrast, plant-based platforms—particularly tobacco—offer several advantages, including well-established genetic transformation protocols, rapid growth, high biomass accumulation, and intrinsic capabilities for complex post-translational modifications and glycosylation (Molina-Hidalgo et al., 2021; Liu et al., 2025).

The progressive elucidation of the ginsenoside biosynthetic pathway has laid the groundwork for its heterologous reconstruction in alternative hosts (Jung et al., 2014; Kim et al., 2015; Park et al., 2016; Hou et al., 2023). Leveraging the 2,3-oxidosqualene precursor naturally produced through tobacco’s native MVA pathway, we introduced three key enzymes catalyzing the biosynthesis of CK and Rh2. We compared production efficiencies between *N. benthamiana* and *N. tabacum* K326, investigated tissue-specific ginsenoside accumulation, and established callus cultures to evaluate the potential for scalable *in vitro* production. Collectively, our study demonstrates the feasibility and efficiency of tobacco as a plant chassis for the heterologous production of rare ginsenosides, providing a strategic framework for future biotechnological and industrial applications.

## 2 Methods

### 2.1 Construction of a multi-gene overexpression vector

Three key enzyme genes involved in the biosynthesis of rare ginsenosides were selected: *dammarenediol synthase* (*PgDDS*, GenBank ID: GU183405.1), *cytochrome P450 CYP716A47* (GenBank ID: JN604536.1), and the *glycosyltransferases UGTPg1* (GenBank ID: KF377585.1) or *UGTPg45* (GenBank ID: KM401918.1). All genes were synthesized by Biorun Co., Ltd (Wuhan, China). Each gene was placed under the control of the cauliflower mosaic virus 35S promoter at the 5′-end and terminated with the *nopaline synthase* (NOS) terminator sequence at the 3′-end. The final construct comprised three expression cassettes arranged in tandem as follows: 35S–*PgDDS*–NOS, 35S–*CYP716A47*–NOS, and 35S–*UGT*–NOS.

### 2.2 Plant transformation

The multi-gene vector was introduced into *Agrobacterium tumefaciens* strain GV3101 via the freeze–thaw method. Transformed cells were cultured in 5 mL YEB medium containing 25 mg/L hygromycin at 28 °C with shaking at 200 rpm until OD_600_ ≈ 0.8. Cells were harvested by centrifugation at 5,000 rpm for 10 min at 4 °C, resuspended in an equal volume of MS medium supplemented with 100 μM acetosyringone, and adjusted to OD_600_ 0.3–0.5. Leaf explants (0.5–1 cm²) from sterile 4–6-week-old *N. benthamiana* and *N. tabacum* K326 plants were pre-cultured on MS medium containing 2.0 mg/L 2,4-D and 0.5 mg/L 6-BA in darkness at 25 °C for 2 days. Explants were immersed in the *Agrobacterium* suspension with gentle shaking for 10 min, blotted dry, and co-cultured on the same medium containing 100 μM acetosyringone in darkness at 20–22 °C for 2–3 days.

After co-cultivation, explants were transferred to decontamination medium (same as pre-culture medium plus 500 mg/L cefotaxime) and incubated in darkness at 25 °C for 7 days. They were then transferred to selection medium (decontamination medium supplemented with 25 mg/L hygromycin) and subcultured every 2 weeks. After 3–4 rounds of subculture, resistant calli were transferred to differentiation medium (MS + 1.0 mg/L 6-BA + 0.2 mg/L NAA + 25 mg/L hygromycin) for 4–6 weeks to induce shoot formation. Shoots of 3–5 cm were moved to rooting medium (½ MS + 0.5 mg/L IBA + 25 mg/L hygromycin) under a 16-hour photoperiod at 25 °C for 4–6 weeks. Once roots developed, plantlets were acclimatized in a greenhouse for 3–5 days and transplanted into sterilized peat soil:perlite (3:1) substrate at 25 °C with 80–90% relative humidity. The primary regenerants obtained from tissue culture were designated as T0 generation. T0 plants were self-pollinated to collect T1 seeds. These T0 plants were grown to maturity and self-pollinated; no further subculturing was involved in generating T1/T2 seeds. T1 seeds were surface-sterilized and germinated on ½ MS medium containing 25 mg/L hygromycin. This germination step was performed only for seedling selection and did not involve callus induction, subculture, or plant regeneration. After 14 days, resistant seedlings were selected and confirmed by PCR for transgene integration. Selected T1 plants were transferred directly to soil (not back to tissue culture) and grown to maturity in the greenhouse under standard conditions, where they were allowed to self-pollinate to produce T2 seeds. Thus, T2 seeds were obtained through seed propagation after two generations of selfing (T0→T1→T2), without any intervening tissue culture subculturing. T2 seeds were harvested, and progeny were germinated on hygromycin-containing medium to identify homozygous lines based on 100% germination rate. T2 plants were used for all subsequent experiments, including ginsenoside quantification, tissue-specific expression analysis, pot experiments, and callus induction. Prior to each experiment, transgene presence in T2 plants was re-confirmed by PCR and RT-PCR.

### 2.3 Identification of transgenic seedlings

Genomic DNA and total RNA were extracted from tobacco seedlings using a rapid plant genomic DNA extraction kit and the GenePure polysaccharide–polyphenol plant RNA rapid extraction kit, respectively. For RNA extraction, on−column DNase I digestion was performed according to the manufacturer’s instructions to eliminate residual genomic DNA contamination. RNA quality and integrity were assessed by NanoDrop spectrophotometry (A260/A280 ratio between 1.9 and 2.1, and A260/A230 ratio > 2.0). For reverse transcription, 1 μg of total RNA was used as template. cDNA was synthesized using oligo(dT) primers and reverse transcriptase according to the following program: 25 °C for 10 min, 50 °C for 60 min, and 85 °C for 5 min to inactivate the enzyme. The resulting cDNA was diluted 5-fold and stored at −20 °C until use. For genomic PCR, 100 ng of purified genomic DNA was used as template. PCR and RT–PCR were performed using primers listed in **Table 1**. The reaction program was as follows: pre-denaturation at 94 °C for 2 minutes; denaturation at 94 °C for 30 s; annealing at 58 °C; extension at 72 °C for 2 minutes 40 s (34 cycles of steps 2–4); and final extension at 72 °C for 5 minutes. Amplified products were analyzed by 0.8% agarose gel electrophoresis.

**Table 1:** Primes of PCR and RT-PCR.

Genes	Primers	Sequences
*Nbactin*	*Nbactin-qF*	5’-AAAGACCAGCTCATCCGTGG-3’
	*Nbactin-qR*	5’-AGCAGCTTCCATTCCGATCA-3’
*actin*	*actin-F*	5’-CGTGATCTTACAGATAGCTTCATGA-3’
	*actin-R*	5’-AGAGAAGCTAAGATTGATCCTCC-3’
*PgDDS*	*PgDDS-F*	5’-ATGTGGAAGCTGAAGGTTGCTCAAGGA-3’
	*PgDDS -R*	5’-TTAAATTTTGAGCTGCTGGTGCTTAGGC-3’
*CYP716A47*	*CYP716A47-F*	5’-ATGGTGTTGTTTTTCTCCCTATCT-3’
	*CYP716A47-R*	5’-TTAATTGTGGGGATGTAGATGAAT-3’
*UGTPg1*	*GT1-F*	5’-ATGAAGTCAGAATTGATATTCTTGCCCG-3’
	*GT1-R*	5’-TTACATTACATAATTTCCTCAAATAGCTT-3’
*UGTPg45*	*GT2-F*	5’-ATGGAGAGAGAAATGTTGAGCAAAACT-3’
	*GT2-R*	5’-TTACATCAGGAGGAAACAAGCTTTGAAA-3’

### 2.4 Topping experiment

T_2_-generation K326 seedlings engineered for CK and Rh2 biosynthesis were grown in 38 × 42 cm cultivation pots at the Fuquan agricultural experiment base (Latitude: 26°42′N, Longitude: 107°31′E; Fuquan City, Guizhou Province, China). At the early bud stage, the plant apex was inspected, and topping was performed by cutting at the base of the 1–2 youngest leaves above the target functional leaves (e.g., to retain 20 functional leaves, cutting was performed at the base of the 21st–22nd small leaves). The cutting height was maintained 2–3 cm above the uppermost functional leaf to avoid injury to the growth point. The apical tissue, including terminal and unopened flower buds, was excised with sterile scissors at a 45° angle to minimize wound size. The excised apex was sealed in a sterile bag and autoclaved prior to disposal to prevent the spread of transgenic material. To protect the wound from infection and hormone loss, the cut surface was covered with sterile plastic film for 1–2 days. Ten days after topping, leaves from both topped and non-topped plants were collected for quantification of CK and Rh2.

### 2.5 Induction and subculture of tobacco callus cells

Seeds of PCR-positive T_2_ transgenic tobacco were surface sterilized with 10% sodium hypochlorite for 10 min, rinsed 4–5 times with sterile water, and sown on MS medium. When seedlings developed 2–3 true leaves, they were transferred to flasks containing MS medium with 25 mg/L hygromycin. After 60 days of growth, leaves and roots were excised under sterile conditions. Leaves were cut into 0.5 × 0.5 cm pieces and cultured on callus induction medium (MS + 1.0 mg/L NAA + 1.0 mg/L 6-BA). Roots were gently scored on both sides with a sterile blade to produce 5–6 small wounds and cultured on root-derived callus induction medium (MS + 1.0 mg/L 2,4-D + 0.5 mg/L NAA + 0.5 mg/L 6-BA). Media were refreshed every two weeks. Callus tissue became visible at the wound edges after 15–25 days and was fully developed after approximately 45 days. Calli were subsequently transferred to subculture medium (MS + 1.0 mg/L NAA + 1.0 mg/L 6-BA) for maintenance and propagation.

### 2.6 Ginsenoside quantification

#### 2.6.1 Sample preparation and LC–MS conditions

Approximately 20 g of fresh tobacco leaves were flash-frozen in liquid nitrogen and lyophilized. The dried tissue was ground into a fine powder using a high-speed grinder. Precisely 0.3 g of powder was weighed into a 15 mL centrifuge tube, and 80% methanol was added at a ratio of 1:29 (g:mL). Ultrasonic extraction was conducted for 2 hours with intermittent shaking. The extract was centrifuged at 15,000 rpm for 10 minutes, and the supernatant was collected for liquid chromatography–mass spectrometry (LC–MS) analysis, which was performed by Novogene Co., Ltd. (Beijing, China) using an AB Sciex QTRAP^®^ 6500+ mass spectrometer coupled with an AB Sciex ExionLC™ AD liquid chromatography system (Framingham, MA, USA).

The concentrations of CK and Rh2 were determined by LC–MS under the following conditions: BEH C18 column (50 × 2.1 mm, 1.7 μm); mobile phases: (A) 0.1% formic acid in water, (B) 0.1% formic acid in methanol; gradient program: 50% B at 0 min → 100% B at 3.5 min → hold at 100% B until 5.2 min → return to 50% B at 5.3 min → hold at 50% B until 6.7 min; column temperature: 40 °C; injection volume: 5 μL; flow rate: 0.4 mL/min. Mass spectrometry was conducted in positive electrospray ionization (ESI^+^) mode with an ion source temperature of 550 °C, voltage of 5,500 V, curtain gas at 40 psi, and both nebulizer and auxiliary gases at 45 psi. Quantitative detection was performed using multiple reaction monitoring (MRM). The optimized MS parameters for each analyte, including precursor ion (Q1), product ion (Q3), retention time (RT), declustering potential (DP), and collision energy (CE), are listed in **Supplementary Table 2**.

#### 2.6.2 Method validation

The LC-MS method was validated for linearity, accuracy, precision, and sensitivity following standard guidelines (**Supplementary Table 2**, **Supplementary Figure S2**). Authentic standards of CK and Rh2 (>98.0% purity) were purchased from Beijing Solarbio Sciences & Technology Co., Ltd. (Beijing, China). Calibration curves were constructed by plotting peak area ratios (analyte/internal standard) against nominal concentrations. For ginsenoside CK, the regression equation was y=0.156x−0.00138 with a correlation coefficient r=0.9993 over a concentration range of 0.05–25 ng/mL. Accuracy at individual calibration points ranged from 94.8% to 103.7%. For ginsenoside Rh2, the regression equation was y=0.225x+0.0403 with r=0.9948 over a range of 1–100 ng/mL. Accuracy ranged from 85.1% to 109.3%; the value at 100 ng/mL (85.1%) meets the lower boundary of the acceptance criterion. Recovery experiments using spiked quality control (QC) samples further confirmed accuracy: for CK at 1.5 ng/mL, recovery was 99.8%; for Rh2 at 30 ng/mL, recovery was 100%. Precision, expressed as relative standard deviation (RSD) of replicate QC samples, was 4% for CK and 2% for Rh2. All values were within the acceptable limits of 85–115% for accuracy and 15% for precision. Based on a signal-to-noise ratio of 3:1, the limit of detection (LOD) was determined to be 0.02 ng/mL for CK and 0.3 ng/mL for Rh2. The limit of quantification (LOQ, S/N ≥ 10) was 0.05 ng/mL for CK and 1.0 ng/mL for Rh2. All sample concentrations measured in this study were above the respective LOQ. Matrix effects were evaluated by comparing analyte peak areas in post−extraction spiked matrix versus neat standards. Extraction recoveries were determined by spiking known amounts of standards before extraction. Both parameters were within acceptable ranges (85–115%), indicating no significant matrix interference.

### 2.7 Statistical analysis

Statistical analyses were performed using Excel 2019 (Microsoft, USA). Data are presented as mean ± SD (standard deviation). One-way ANOVA followed by Tukey’s HSD post-hoc test was used for multiple comparisons, and Student’s t-test was used for two-group comparisons. All experiments were performed with three biological replicates per line (three independently grown plants). For RT-qPCR analysis, three technical replicates were included for each biological replicate. For LC-MS quantification, each biological replicate was analyzed once; technical replicates were not performed because the LC-MS method was thoroughly validated (Section 2.6.2) and biological triplicates provided adequate statistical power. Exact p values for all pairwise comparisons are available from the corresponding author upon request.

## 3 Results

### 3.1 Vector construction and screening of transgenic lines for heterologous synthesis of rare ginsenoside CK in tobacco

**Figure 1:**
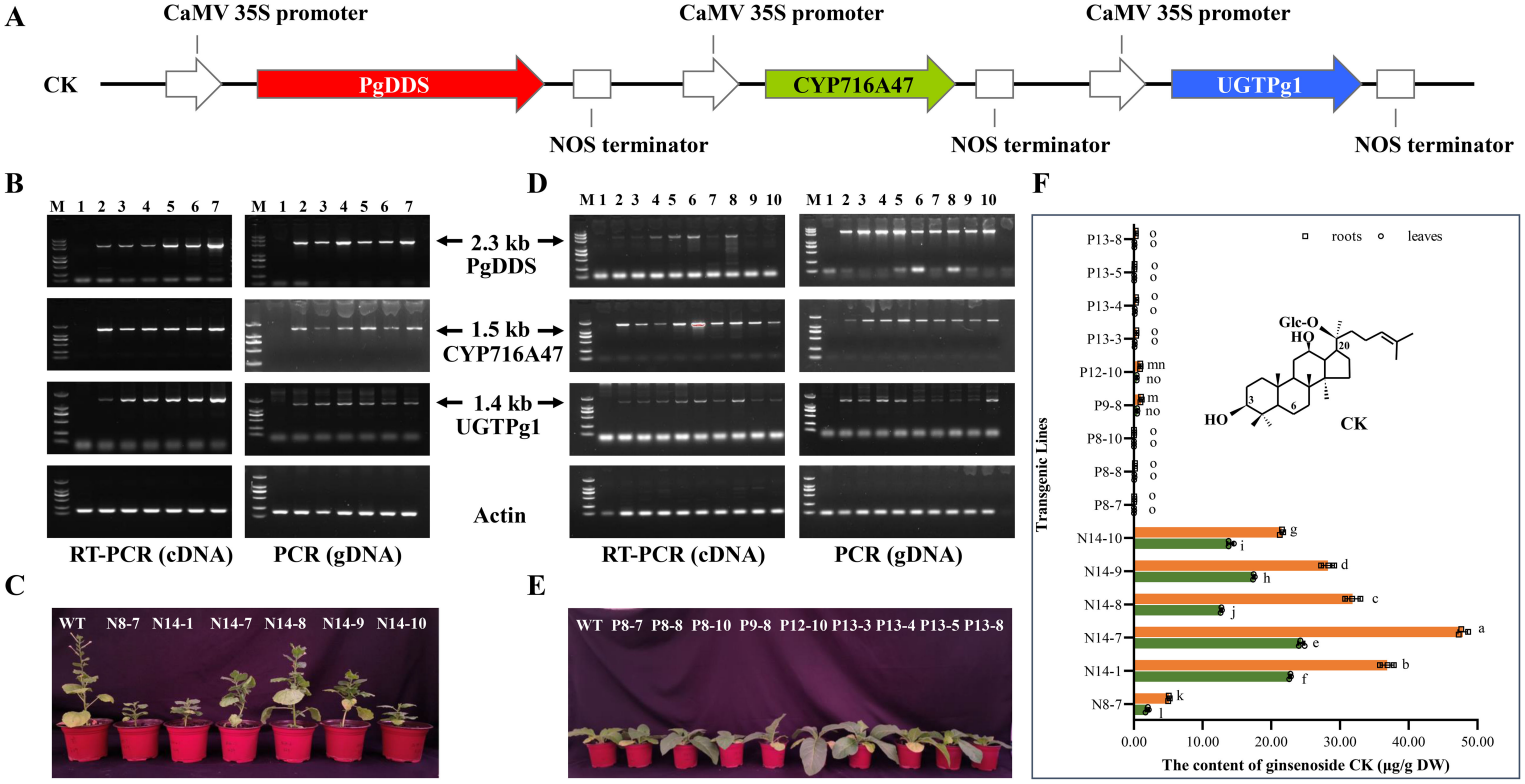
Heterologous synthesis of rare ginsenoside CK in *N. benthamiana* and K326. **(A)** Schematic diagram of the vector for synthesizing rare ginsenoside CK; **(B)** Screening of positive lines synthesizing rare ginsenoside CK in *N. benthamiana* by PCR and RT-PCR. Lane M: DNA marker, Lane 1: WT (untreated *N. benthamiana*), Lane 2: Line N8-7, Lane 3: Line N14-1, Lane 4: Line N14-7, Lane 5: Line N14-8, Lane 6: Line N14-9, Lane 7: Line N14-10; **(C)** Screened positive lines of *N. benthamiana*; **(D)** Screening of positive lines synthesizing rare ginsenoside CK in K326 by PCR and RT-PCR. Lane M: DNA marker, Lane 1: WT (untreated K326), Lane 2: Line P8-7, Lane 3: Line P8-8, Lane 4: Line P8-10, Lane 5: Line P9-8, Lane 6: Line P12-10, Lane 7: Line P13-3, Lane 8: Line P13-4, Lane 9: Line P13-5, Lane 10: Line P13-8; **(E)** Screened positive lines of K326; **(F)** Detection of rare ginsenoside CK content in positive lines. Data are presented as mean ± SD (n = 3 biological replicates per line). Individual data points are shown as dots. Different letters indicate significant differences (P < 0.05, one−way ANOVA with Tukey’s HSD post−hoc test). Exact p values for pairwise comparisons are available from the corresponding author upon request. Note: Different letters indicate significant differences. The DNA marker used in PgDDS gene detection is DL5000 from UEland Company, which consists of 8 double-stranded DNA fragments with sizes of 5,000 bp, 3,000 bp, 2,000 bp, 1,000 bp, 750 bp, 500 bp, 250 bp, and 100 bp. For other types of gene detection, the DNA marker employed is DL2000 from UEland Company, composed of 6 double-stranded DNA fragments with lengths of 2,000 bp, 1,000 bp, 750 bp, 500 bp, 250 bp, and 100 bp. PCR was performed on genomic DNA or RT-PCR on cDNA (1B, D).

The multi-gene overexpression vector containing the *UGTPg1* gene was successfully introduced into *N. benthamiana* and K326 (**Figure 1A**). Following systematic screening of 140 N*. benthamiana* transgenic lines, six positive lines capable of synthesizing the rare ginsenoside CK were identified: N8-7, N14-1, N14-7, N14-8, N14-9, and N14-10 (**Figures 1B, C**; **Supplementary Table 1**). Metabolite quantification revealed that the maximum CK content in leaves reached 24.36 μg/g DW, whereas roots accumulated up to 47.87 μg/g DW. In all lines, CK levels were consistently higher in roots than in leaves, with root-to-leaf ratios ranging from 1.53 to 2.59 (**Figure 1F**).

Screening of 130 transgenic K326 lines identified nine CK-producing lines: P8-7, P8-8, P8-10, P9-8, P12-10, P13-3, P13-4, P13-5, and P13-8 (**Figure 1D, E**; **Supplementary Table 1**). However, CK accumulation in the K326 background was notably lower, with maximum concentrations of 0.49 μg/g DW in leaves and 1.04 μg/g DW in roots. As observed in *N. benthamiana*, CK levels were higher in roots than in leaves, with root-to-leaf ratios ranging from 1.52 to 3.26 (**Figure 1F**).

Comparison of identical tissues across the two tobacco species showed that CK accumulation was significantly higher in transgenic *N. benthamiana* than in K326 under our experimental conditions, suggesting that *N. benthamiana* may be a more suitable host for CK production, though this conclusion is tempered by event-to-event expression variability (see Discussion).

### 3.2 Vector construction and screening of transgenic lines for heterologous synthesis of rare ginsenoside Rh2 in tobacco

**Figure 2:**
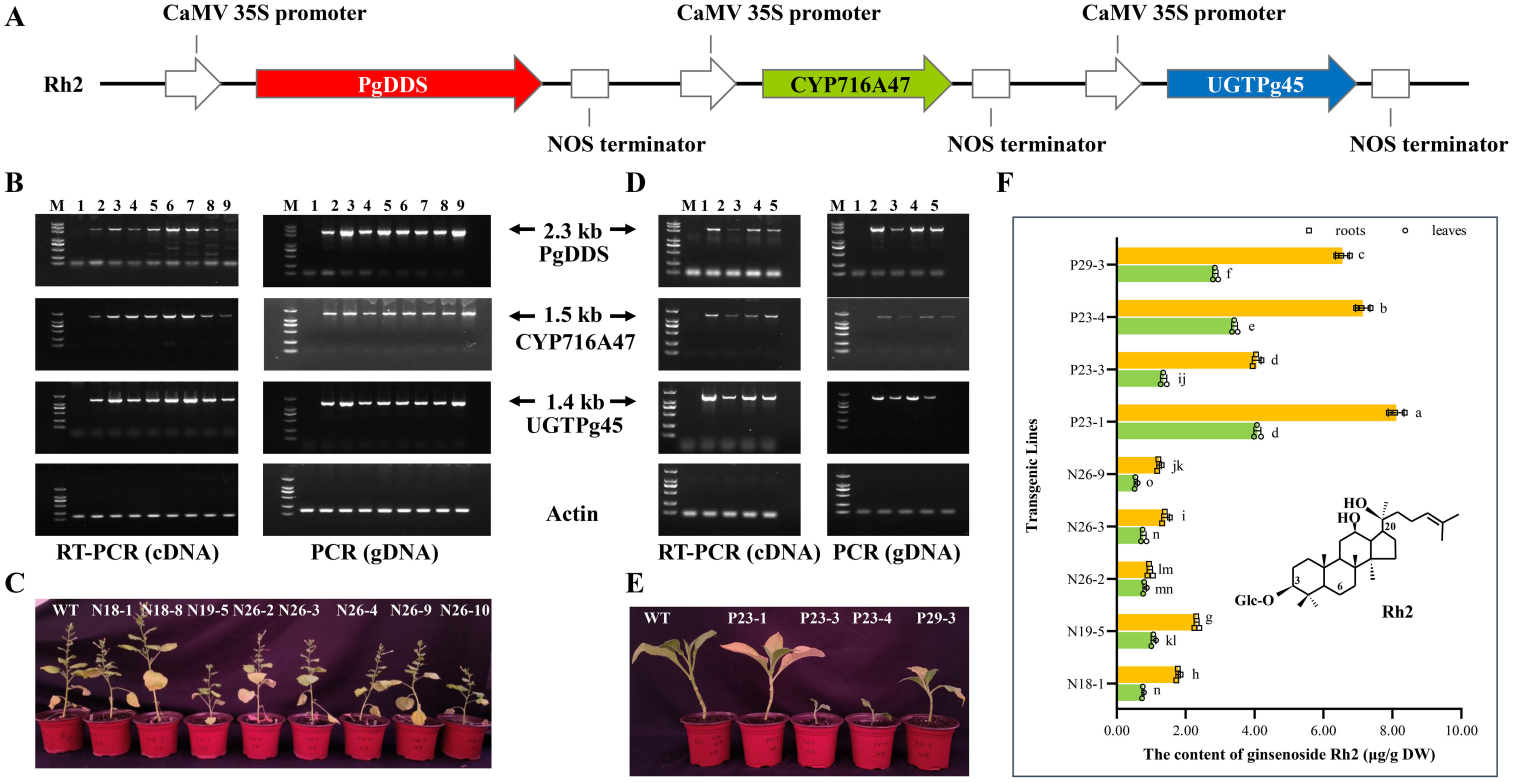
Heterologous synthesis of rare ginsenoside Rh2 in *N. benthamiana* and K326. **(A)** Schematic diagram of the vector for synthesizing rare ginsenoside Rh2; **(B)** Screening of positive lines synthesizing rare ginsenoside Rh2 in *N. benthamiana* by PCR and RT-PCR. Lane M: DNA marker, Lane 1: WT (untreated *N. benthamiana*), Lane 2: Line N18-1, Lane 3: Line N18-8, Lane 4: Line N19-5, Lane 5: Line N26-2, Lane 6: Line N26-3, Lane 7: Line N26-4, Lane 8: Line N26-9, Lane 9: Line N26-10; **(C)** Screened positive lines of *N. benthamiana*; **(D)** Screening of positive lines synthesizing rare ginsenoside Rh2 in K326 by PCR and RT-PCR. Lane M: DNA marker, Lane 1: WT (untreated K326), Lane 2: Line P23-1; Lane 3: Line P23-3; Lane 4: Line P23-4; Lane 5: Line P29-3; **(E)** Screened positive lines of K326; **(F)** Detection of rare ginsenoside Rh2 content in positive lines. Data are presented as mean ± SD (n = 3 biological replicates per line). Individual data points are shown as dots. Different letters indicate significant differences (P < 0.05, one−way ANOVA with Tukey’s HSD post−hoc test). Exact p values for pairwise comparisons are available from the corresponding author upon request. Note: Different letters indicate significant differences. The usage of the DNA marker is the same as that shown in **Figure 1**. PCR was performed on genomic DNA or RT-PCR on cDNA (2B, D).

The multi-gene overexpression vector harboring the *UGTPg45* gene was introduced into *N. benthamiana* and K326 (**Figure 2A**). Screening of 130 N*. benthamiana* transgenic lines identified eight Rh2-producing lines: N18-1, N18-8, N19-5, N26-2, N26-3, N26-4, N26-9, and N26-10 (**Figures 2B, C**; **Supplementary Table 1**). Metabolite analysis revealed that Rh2 levels in these lines reached 1.07 μg/g DW in leaves and 2.32 μg/g DW in roots. In productive lines, Rh2 content was consistently higher in roots than in leaves, with root-to-leaf ratios of 1.18–2.35. Notably, Rh2 was undetectable in lines N18-8, N26-4, and N26-10 (**Figure 2F**).

Screening of 170 transgenic K326 lines yielded four Rh2-producing lines: P23-1, P23-3, P23-4, and P29-3 (**Figures 2D, E**; **Supplementary Table 1**). Rh2 accumulation in K326 was substantially higher than in *N. benthamiana*, with maximum concentrations of 4.08 μg/g DW in leaves and 8.11 μg/g DW in roots. As with *N. benthamiana*, Rh2 content in K326 roots exceeded that in leaves by 1.99–2.99 times (**Figure 2F**).

When comparing equivalent tissues between the two species, transgenic K326 showed markedly higher Rh2 accumulation than *N. benthamiana* in the lines we analyzed, suggesting that K326 may be a more suitable host for Rh2 production, but this should be interpreted with caution due to potential line-specific effects.

### 3.3 Pot experiments with transgenic K326 lines producing rare ginsenosides

**Figure 3:**
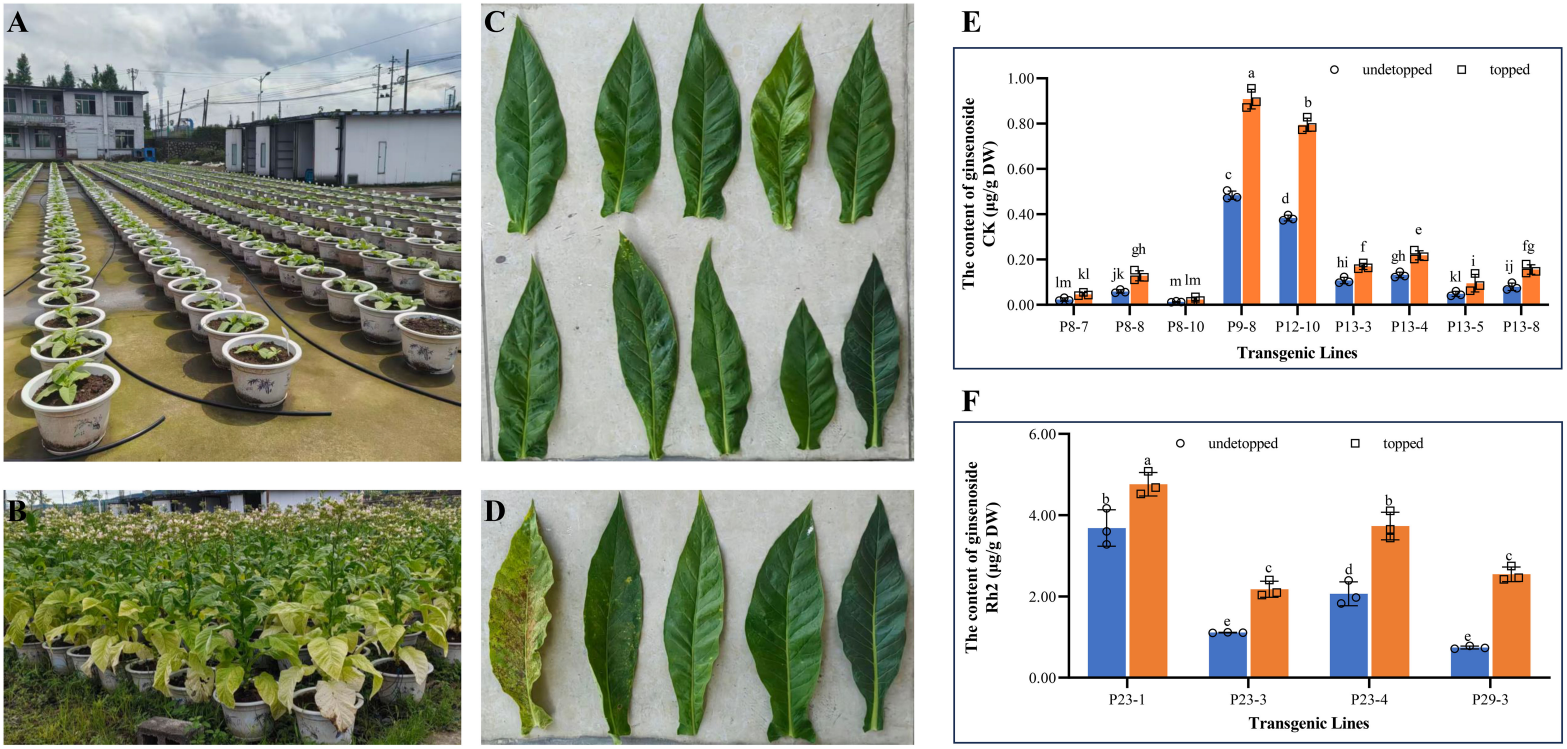
Pot experiment of transgenic K326 lines synthesizing rare ginsenosides CK and Rh2. **(A)** Pot-cultured seedling stage; **(B)** Pot-cultured full flowering stage; **(C)** Upper-middle leaves of transgenic K326 lines synthesizing rare ginsenoside CK. In the first row, from left to right: P8-7, P8-8, P8-10, P9-8, P12-10; In the second row, from left to right: P13-3, P13-4, P13-5, P13-8, K326 (control); **(D)** Upper-middle leaves of transgenic K326 lines synthesizing rare ginsenoside Rh2. From left to right: P23-1, P23-3, P23-4, P29-3, K326 (control); **(E, F)** Effect of topping (apical bud removal) on ginsenoside accumulation in transgenic K326 lines. **(E)** Ginsenoside CK content in CK−producing lines; **(F)** Ginsenoside Rh2 content in Rh2−producing lines. Data are presented as mean ± SD (n = 3 biological replicates per treatment). Individual data points are shown as dots. Different letters indicate significant differences (P < 0.05, Student’s t−test). Exact p values are available from the corresponding author upon request. Different letters indicate significant differences.

To investigate the effect of C apical bud removal (topping) on ginsenoside accumulation, we compared topped and non-topped plants of selected transgenic K326 lines (**Figures 3A, B**). At full flowering, CK-producing K326 lines remained phenotypically similar to the control (**Figure 3C**), while Rh2-producing lines developed more severe weather flecking (**Figure 3D**). Ten days after topping, both CK and Rh2 levels were higher in topped plants than in non-topped controls under our experimental conditions. Specifically, in CK-producing lines, topped plants showed 1.58–2.18 times higher CK content, with a maximum of 0.91 μg/g DW in line P9-8 (**Figure 3E**). In Rh2-producing lines, topped plants exhibited 1.29–3.43 times higher Rh2 levels, with a maximum of 4.76 μg/g DW (**Figure 3F**). However, these results should be interpreted cautiously, as the experiment lacked mock-wounding controls and did not account for potential differences in plant developmental stage or biomass changes between topped and non-topped groups. Whether the observed increase is specifically due to altered source–sink relationships following apical dominance removal or merely reflects a general wound stress response remains to be determined in future studies with more rigorous experimental designs (e.g., time-course sampling, wounding controls, and calculation of total content per plant).

### 3.4 Acquisition and content detection of *N. benthamiana* cells synthesizing rare ginsenosides

**Figure 4:**
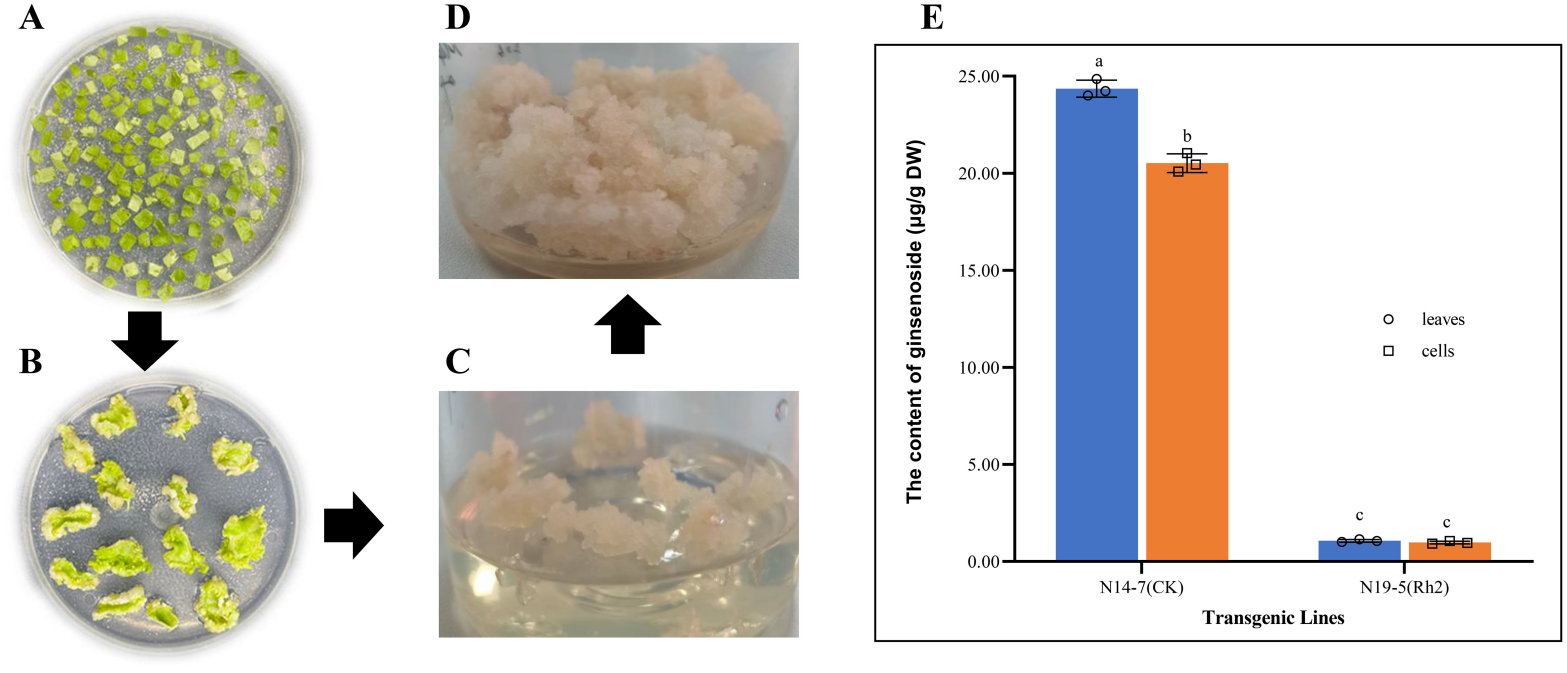
Heterologous synthesis of rare ginsenosides CK and Rh2 in tobacco cells. **(A)** Dedifferentiation treatment of leaves; **(B)** Induction of callus cells; **(C)** Acquisition of callus cells; **(D)** Culture of callus cells; **(E)** Detection of saponin content in cells. Data are presented as mean ± SD (n = 3 biological replicates per line). Individual data points are shown as dots. Different letters indicate significant differences (P < 0.05, one−way ANOVA with Tukey’s HSD post−hoc test). Exact p values are available from the corresponding author upon request. Different letters indicate significant differences.

Callus cells were successfully induced from the leaves and roots of transgenic *N. benthamiana* lines capable of synthesizing rare ginsenosides through dedifferentiation culture (**Figures 4A-D**). In callus cells derived from leaves, the contents of ginsenoside CK and Rh2 were 20.52 μg/g DW and 0.98 μg/g DW, respectively. The CK level was slightly but significantly lower than that in the leaves of transgenic *N. benthamiana*, whereas the Rh2 content showed no significant difference (**Figure 4E**).

In contrast, root-derived callus exhibited pronounced browning, which markedly impaired cellular vitality and subsequent growth (**Supplementary Figure S1**). During culture, the callus gradually accumulated brown pigments, altering cell morphology and inhibiting proliferation. As a result, an adequate quantity of root-derived callus cells for ginsenoside detection has not yet been obtained.

## 4 Discussion

### 4.1 Overall performance of tobacco as a heterologous chassis for rare ginsenosides and promoter competition

Plant metabolic engineering has advanced rapidly, enabling the development of sustainable platforms for producing high-value phytochemicals. Compared with microbial systems, which often face challenges such as high cultivation costs, contamination risks, and the inability to replicate complex plant glycosylation patterns (Li et al., 2023), plant chassis provide unique advantages, including robust transformation protocols, native post-translational modification capacity, and scalability for biomass-based production (Wang et al., 2025). In this study, tobacco species (*N. benthamiana* and K326) were engineered to biosynthesize CK and Rh2 by introducing only three core biosynthetic genes—*DDS*, *CYP716A47*, and either *UGTPg1* or *UGTPg45*. This streamlined pathway demonstrates the capacity of plant systems to leverage endogenous metabolism, minimizing the need for extensive synthetic reconstruction (Wu et al., 2021; Abdulhafiz et al., 2022; Khalafalla, 2026).

Previous studies have achieved the heterologous production of rare ginsenosides in tobacco through multi-gene expression. Gwak et al. introduced *PgDDS*, *CYP716A47*, and *UGT71A28* from *Panax ginseng*, producing CK with the highest content of 4.65 μg/g DW in roots (Gwak et al., 2017). Similarly, Chen et al. reconstructed the biosynthetic pathway for Rh2 in tobacco using *PnDDS*, *CYP12H*, and *UGTPn3* from *P. notoginseng*, achieving a maximum root content of 5.30 μg/g DW (Chen et al., 2023). In the present study, CK accumulation in *N. benthamiana* roots reached 47.87 μg/g DW, and Rh2 content in K326 roots reached 8.11 μg/g DW—both substantially exceeding previous reports.

However, these high yields were achieved despite a potential technical limitation in our construct design: all three biosynthetic genes (PgDDS, CYP716A47, and UGTPg1/UGTPg45) were driven by the same CaMV 35S promoter in a tandem-arranged multigene vector. This design raises legitimate concerns regarding transcriptional interference, promoter competition, and gene silencing, which may lead to unbalanced expression among transgenes and contribute to line-to-line variability (Butaye et al., 2005; Venter, 2007). Indeed, repeated use of identical strong promoters in plant transformation vectors has been reported to trigger homology-dependent gene silencing, particularly when multiple copies of the same promoter are present in the T-DNA region (Matzke and Matzke, 1995; De Buck et al., 2000). Despite this limitation, we chose the tandem 35S configuration for its simplicity and proven efficacy in numerous plant metabolic engineering studies, including previous heterologous production of ginsenosides in tobacco (Gwak et al., 2017; Chen et al., 2023). The successful generation of transgenic lines with measurable CK and Rh2 levels—substantially exceeding previous reports—indicates that promoter competition did not completely abolish transgene expression. Future improvements could adopt alternative strategies to mitigate promoter competition: (1) using different promoters (e.g., Ubiquitin, Actin, or tissue-specific promoters) for each gene (Koska et al., 2025); (2) employing polycistronic constructs mediated by 2A peptides or internal ribosome entry sites (IRES) to express multiple genes from a single promoter (Gouiaa et al., 2012; Liu et al., 2017); or (3) applying site-specific integration to ensure predictable transgene expression (Anand et al., 2019; Yan et al., 2025). These approaches would help achieve more balanced expression and reduce line-to-line variation in future metabolic engineering efforts.

### 4.2 Host preference and its limitations

A key finding of this study is the distinct host preference for different ginsenosides. *N. benthamiana* demonstrated superior CK production, with root content (47.87 μg/g DW) greatly surpassing that of K326 (1.04 μg/g DW). Conversely, K326 was more efficient for Rh2 synthesis, producing up to 8.11 μg/g DW in roots compared to only 2.32 μg/g DW in *N. benthamiana*. These species-specific disparities underscore the necessity of tailored chassis selection for optimal metabolite yields. Such differences likely arise from variations in metabolic flux distribution, glycosylation efficiency, and the compatibility between heterologous enzymes and endogenous host metabolism (Shih and Morgan, 2020). Consistent with this notion, a comparative metabolomic study of *N. benthamiana* and *N. tabacum* revealed that before flower emergence, N*. benthamiana* exhibits increased central carbon metabolism and higher amino acid levels, potentially providing greater precursor availability for heterologous metabolite biosynthesis (Drapal et al., 2021). Moreover, tissue-specific transport and storage mechanisms—particularly root sequestration of triterpenoids—may further influence compound accumulation (Dong and Qi, 2025). This phenomenon parallels examples in plant metabolic engineering, such as the preferential use of *Artemisia annua* for artemisinin production (Zhao et al., 2022). Consequently, precise host selection is a crucial determinant of efficiency in heterologous ginsenoside biosynthesis. However, direct cross-species comparisons using independently transformed lines are complicated by several confounding variables. Transgene expression in plants is known to vary substantially due to differences in integration locus, copy number, and the occurrence of homology-dependent gene silencing, all of which can influence final metabolite yields independently of the host species’ intrinsic metabolic capacity (Peach and Velten, 1991; Matzke and Matzke, 1995). While we performed RT-qPCR analysis of the three introduced genes in representative high-accumulating lines of both species to help address this concern (**Supplementary Figure 3**), we cannot fully exclude the possibility that event-specific expression levels contributed to the observed differences due to the large number of lines screened and the difficulty of obtaining isogenic backgrounds across two different species. Accordingly, our conclusion that *N. benthamiana* is a better chassis for CK and K326 for Rh2 should be interpreted as a preliminary observation based on the best-performing lines under identical experimental conditions; future studies employing site-specific integration systems or larger panels of independently transformed lines are needed to rigorously establish host preference (Ow, 2011; Yan et al., 2025). Furthermore, we did not determine transgene copy number or insertion locus for the transgenic lines used in this study. Both factors are known to influence transgene expression levels and can contribute to line−to−line variability independently of the host species’ intrinsic metabolic capacity (Peach and Velten, 1991; Ow, 2011). Therefore, our conclusion regarding host preference should be considered as an observational finding that requires validation in lines with controlled copy number and defined insertion sites, for example through site−specific integration.

### 4.3 Topping treatment and weather flecking observation

The observed increase in CK and Rh2 levels after topping, while consistent across multiple lines, should be considered preliminary. The lack of a mock-wounded control (e.g., cutting leaf tips instead of removing the apex) makes it impossible to dissect whether the effect is specifically attributable to the removal of apical dominance or simply a general wound response. Furthermore, because plants were not matched for developmental stage at the time of topping and were sampled only once after 10 days, we cannot rule out that differences in growth rate or leaf age contributed to the apparent yield differences. Future experiments should include time-course analyses, wounding controls, and measurements of total compound accumulation per plant to validate and extend these observations. Additionally, an interesting physiological observation was the pronounced weather flecking in Rh2-producing K326 lines, especially P23-1, the highest-accumulating line. This phenotype, characterized by necrotic lesions and premature senescence, superficially resembles oxidative injury often associated with ozone stress. One possible interpretation is that excessive Rh2 accumulation could impose a metabolic burden, potentially competing for ATP and NADPH and thereby affecting redox homeostasis (Sood, 2025). Additionally, Rh2 has been reported to have cytotoxic properties and to induce apoptosis through ROS-mediated mechanisms in mammalian cells (Liu et al., 2021). However, the current data show only a correlation between high Rh2 levels and leaf necrosis; they do not establish causation. Alternative explanations cannot be excluded, including line-specific insertion effects (position effects or transgene copy number), general metabolic burden from overexpression of multiple foreign genes, differences in plant developmental stage, or subtle environmental variation (e.g., localized ozone exposure) that may have coincided with the phenotype (Baslam et al., 2020). Therefore, while the association is intriguing, we refrain from concluding that Rh2 directly causes the observed weather flecking. Future studies will be required to test causality more rigorously, for example by using inducible expression systems to temporally control Rh2 production, measuring ROS levels and lipid peroxidation in the same genetic background with multiple independent lines, or applying exogenous Rh2 to wild-type leaves. Until such evidence is available, the weather flecking phenotype should be regarded as a correlative observation that warrants further investigation.

### 4.4 Callus culture: proof−of−concept and current limitations

The establishment of transgenic tobacco callus capable of ginsenoside synthesis provides a proof-of-concept foundation for further development. While current yields in tobacco callus (20.52 μg/g DW for CK) are substantially lower than those achieved in top-performing microbial systems (Yan et al., 2014; Zhuang et al., 2017; Li et al., 2019; Wang et al., 2019), plant cell cultures offer certain inherent advantages, such as the ability to functionally express plant cytochrome P450s and to perform complex glycosylation patterns without extensive pathway re-engineering. However, at this stage, our callus data alone do not support claims of scalability or industrial competitiveness. Significant technical hurdles remain, including the relatively low product titers, the browning problem observed in root derived callus, and the absence of suspension culture optimization, bioreactor studies, or long term stability assessments. Future work will be required to determine whether tobacco cell suspensions can be developed into a truly scalable platform, e.g., by improving gene expression, enhancing precursor supply, applying elicitors, preventing browning, and transitioning from solid callus to liquid suspension cultures in bioreactors (Shaaltiel et al., 2007; Appelhagen et al., 2018). Until such studies are performed, the callus system should be regarded as a preliminary demonstration of heterologous ginsenoside production in tobacco cells, not as a ready−to−use production platform.

In this study, transgenic *N. benthamiana* leaf-derived calli showed robust proliferation, while root-derived calli displayed severe browning immediately after induction. Root callus browning was coupled with reduced cell viability and inhibited cell growth. This prevented accurate ginsenoside quantification and limited the establishment of scalable root suspension culture platforms. Tissue browning is mainly caused by wound-induced phenolic oxidation catalyzed by endogenous polyphenol oxidases (PPOs) and peroxidases (PODs) (Xu et al., 2015; Permadi et al., 2024). The generated quinone derivatives form toxic melanin-like pigments, trigger oxidative stress, and suppress cell proliferation (Permadi et al., 2024). The severe browning phenotype in root calli is largely due to the inherently higher phenolic contents and antioxidant enzyme activities in root tissues compared to leaves (Kang et al., 2025). Moreover, heterologous ginsenoside biosynthesis may further aggravate membrane stress, rendering root calli more susceptible to browning. Several feasible strategies can be applied to alleviate this challenge in future studies, including exogenous antioxidant supplementation (e.g., ascorbic acid, citric acid, or polyvinylpyrrolidone), low-temperature dark incubation, optimization of auxin regimens, and screening of high-quality explants or browning-resistant genotypes (Dong et al., 2016; Permadi et al., 2024). Overall, the leaf callus system validated the feasibility of heterologous ginsenoside production in tobacco. Further optimization of root callus culture protocols is a prerequisite for achieving stable, large-scale, and season-independent biomanufacturing of rare ginsenosides.

## 5 Conclusions

This study successfully engineered tobacco plants and cell cultures for the heterologous production of rare and pharmaceutically valuable ginsenosides CK and Rh2. Stable T2-generation transgenic lines of *N. benthamiana* and K326 were generated, each capable of synthesizing the target compounds, thereby confirming the feasibility of producing rare ginsenosides in tobacco through metabolic engineering. Root tissues served as the primary sites of ginsenoside accumulation, with concentrations consistently exceeding those in leaves, underscoring the critical role of roots in triterpenoid storage. Our results suggest a possible species specific preference: under our experimental conditions, *N. benthamiana* showed higher ginsenoside CK accumulation, whereas K326 showed higher Rh2 accumulation. However, direct cross-species comparisons are complicated by event-to-event expression variability, and additional studies are needed to rigorously establish host preference. Topping (apical bud removal) enhanced CK and Rh2 levels in K326 under our experimental conditions, but the lack of mock-wounding controls and time-course sampling means that these findings should be considered preliminary. The successful establishment of ginsenoside-producing callus cultures provides a proof-of-concept for future scale-up efforts, though current yields remain low and browning of root-derived callus must be addressed. A correlative observation of weather flecking in high-Rh2-accumulating lines does not imply causation and requires further mechanistic investigation.

Despite these limitations, our work establishes tobacco as a promising plant chassis for rare ginsenoside production and provides a foundation for future optimization strategies, including promoter diversification, pathway balancing, and improved culture conditions.

## Data Availability

The datasets presented in this study can be found in online repositories. The names of the repository/repositories and accession number(s) can be found in the article/**Supplementary Material**.

## Supplementary material

Supplementary Figure 1 Leaves and root callus cells of *N. benthamiana.* **(A)** Induction of leaves callus cells; **(B)** Induction of roots callus cells; **(C)** Acquisition and culture of leaf-derived callus cells; **(D)** Acquisition and culture of root-derived callus cells.

Supplementary Figure 2 LC-MS chromatograms of standard curves and test samples for CK and Rh2. **(A)** Standard curves of ginsenoside CK determined by LC-MS; **(B)** Standard curves of ginsenoside Rh2 determined by LC-MS; **(C)** Peak review of ginsenoside CK in partial samples by LC-MS; **(D)** Peak review of ginsenoside Rh2 in partial samples by LC-MS.

Supplementary Figure 3 Relative transcript levels of the three introduced genes in representative transgenic tobacco lines. Total RNA was extracted from leaves of T2 transgenic lines. Transcript levels of PgDDS, CYP716A47, and the respective glycosyltransferase gene (UGTPg1 for CK−producing lines; UGTPg45 for Rh2−producing lines) were measured by RT−qPCR and normalized to the endogenous Actin gene. Values are presented as fold change relative to the lowest−expressing line within each gene group (set to 1.0) to enable comparison across lines. Bars represent means ± SD from three biological replicates. (A) CK−producing lines: *N. benthamiana* (N8−7, …, N14−10) and (B) K326 (P8−7, …., P13−8). (C) Rh2−producing lines: *N. benthamiana* (N18-1, …., N26−9) and (D)K326 (P23−1, …, P29−3). Note that wild−type plants lack these transgenes and therefore cannot serve as a baseline; instead, the lowest expressing line within each panel was used as calibrator. Different letters above bars indicate significant differences among lines (one−way ANOVA with Tukey’s post−hoc test).

Supplementary Table 1 Transgenic Positive Tobacco Lines for Heterologous Synthesis of Rare Ginsenosides CK and Rh2.

Supplementary Table 2 LC-MS method validation parameters.

Supplementary Table 3 Primer sequences used for RT−qPCR analysis.
