# Supplementary material for: Metabolic engineering of tobacco for heterologous production of rare ginsenosides CK and Rh2
Source: Front Plant Sci. 2026 Jul 7;17:1884201. doi: 10.3389/fpls.2026.1884201 (PMC13385675; doi:10.3389/fpls.2026.1884201)
Supplement: Supplementary Figure 1 — Leaves and root callus cells of N. benthamiana. (A) Induction of leaves callus cells; (B) Induction of roots callus cells; (C) Acquisition and culture of leaf-derived callus cells; (D) Acquisition and culture of root-derived callus cells. [file DataSheet1.pdf]

A

Analyte Name: Ginsenoside CK-1
Internal Standard: Repaglinide

Data File	2024072002.wiff	Result Table	2024072001.rdb
Acquisition Date	7/20/2024 7:16:57 PM	Algorithm Used	MQL
Acquisition Method	Dam-Pro-Oxi-GinCK-GinRh2-Cyc-LCMS-001.dam	Instrument Name	Sciex Triple Quad 6500+
Project	HC06		

Regression Equation: y = 0.156 x + -0.00138 (r = 0.9993)

Expected Concentration	Number of Values	Mean Calculated Concentration	% Accuracy	Std. Deviation	%CV
0.05	1	0.05	101.3	NaN	NaN
0.1	1	0.10	100.3	NaN	NaN
0.25	1	0.24	94.8	NaN	NaN
0.5	1	0.48	95.7	NaN	NaN
1.5	1	1.50	99.8	NaN	NaN
5	1	5.16	103.1	NaN	NaN
10	1	10.13	101.3	NaN	NaN
25	1	25.92	103.7	NaN	NaN

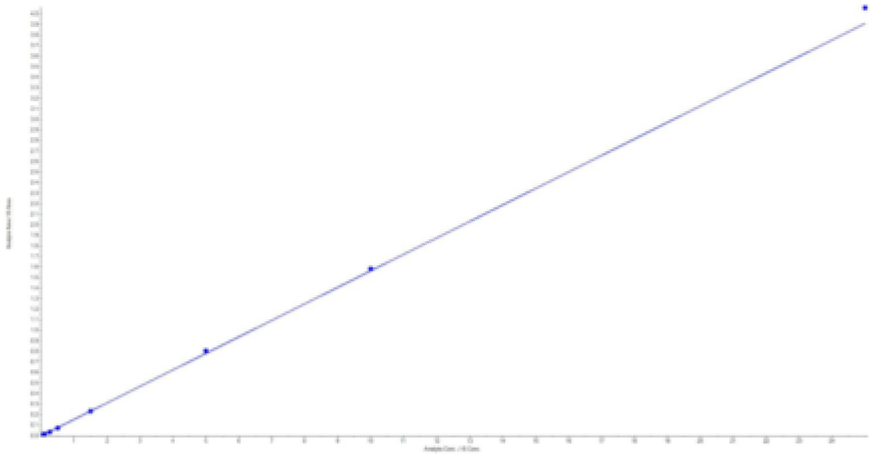

Standard curves of ginsenoside CK determined by LC-MS

B

Analyte Name: Ginsenoside Rh2-1
Internal Standard: Repaglinide

Data File	2024072002.wiff	Result Table	2024072001.rdb
Acquisition Date	7/20/2024 7:16:57 PM	Algorithm Used	MQL
Acquisition Method	Dam-Pro-Oxi-GinCK-GinRh2-Cyc-LCMS-001.dam	Instrument Name	Sciex Triple Quad 6500+
Project	HC06		

Regression Equation: y = 0.225 x + 0.0403 (r = 0.9948)

Expected Concentration	Number of Values	Mean Calculated Concentration	% Accuracy	Std. Deviation	%CV
1	1	0.97	96.9	NaN	NaN
2	1	2.03	101.7	NaN	NaN
5	1	5.46	109.3	NaN	NaN
10	1	10.53	105.3	NaN	NaN
30	1	30.51	101.7	NaN	NaN
100	1	85.10	85.1	NaN	NaN

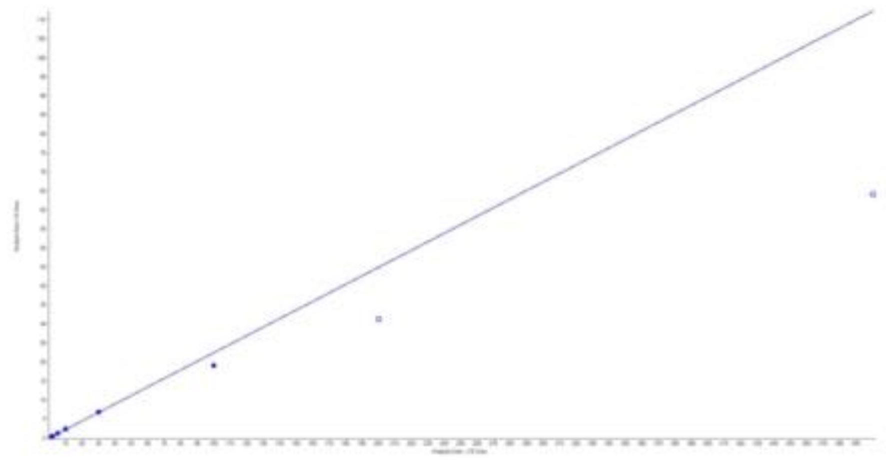

Standard curves of ginsenoside Rh2 determined by LC-MS

C

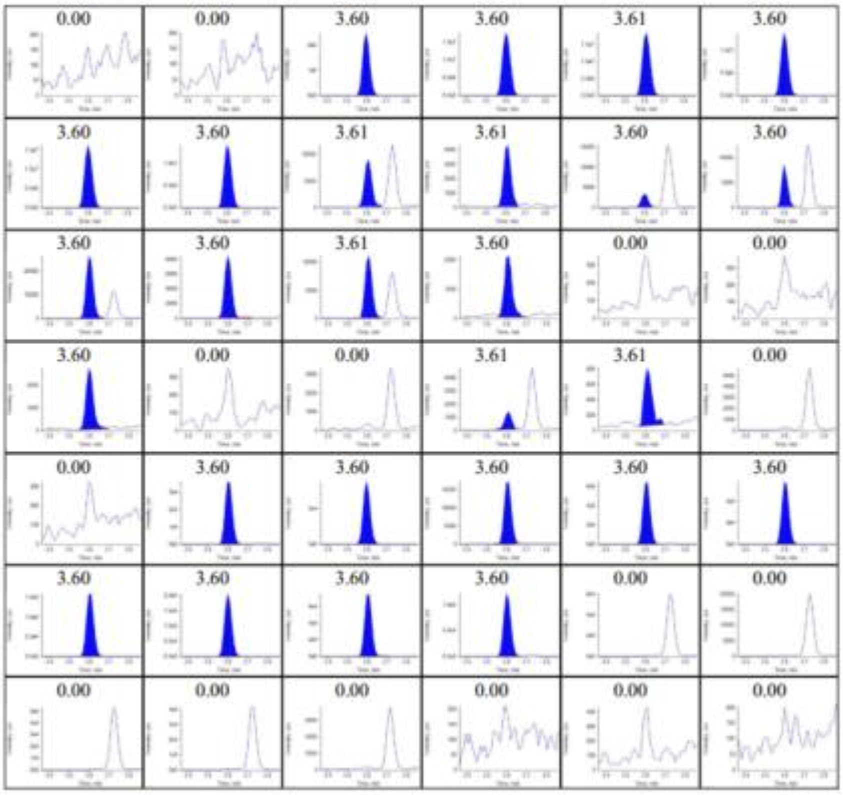

Peak review of ginsenoside CK in partial samples by LC-MS

D

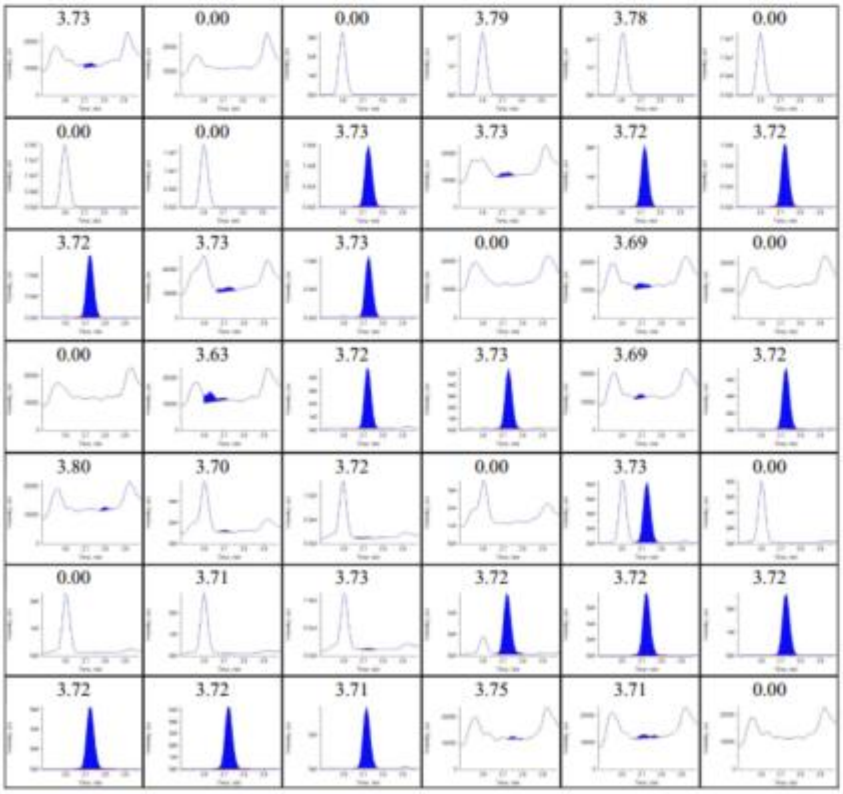

Peak review of ginsenoside Rh2 in partial samples by LC-MS
