# Supplementary material for: Metabolic engineering of tobacco for heterologous production of rare ginsenosides CK and Rh2
Source: Front Plant Sci. 2026 Jul 7;17:1884201. doi: 10.3389/fpls.2026.1884201 (PMC13385675; doi:10.3389/fpls.2026.1884201)
Supplement: Supplementary file 3 [file Presentation1.pptx]

## Slide 1
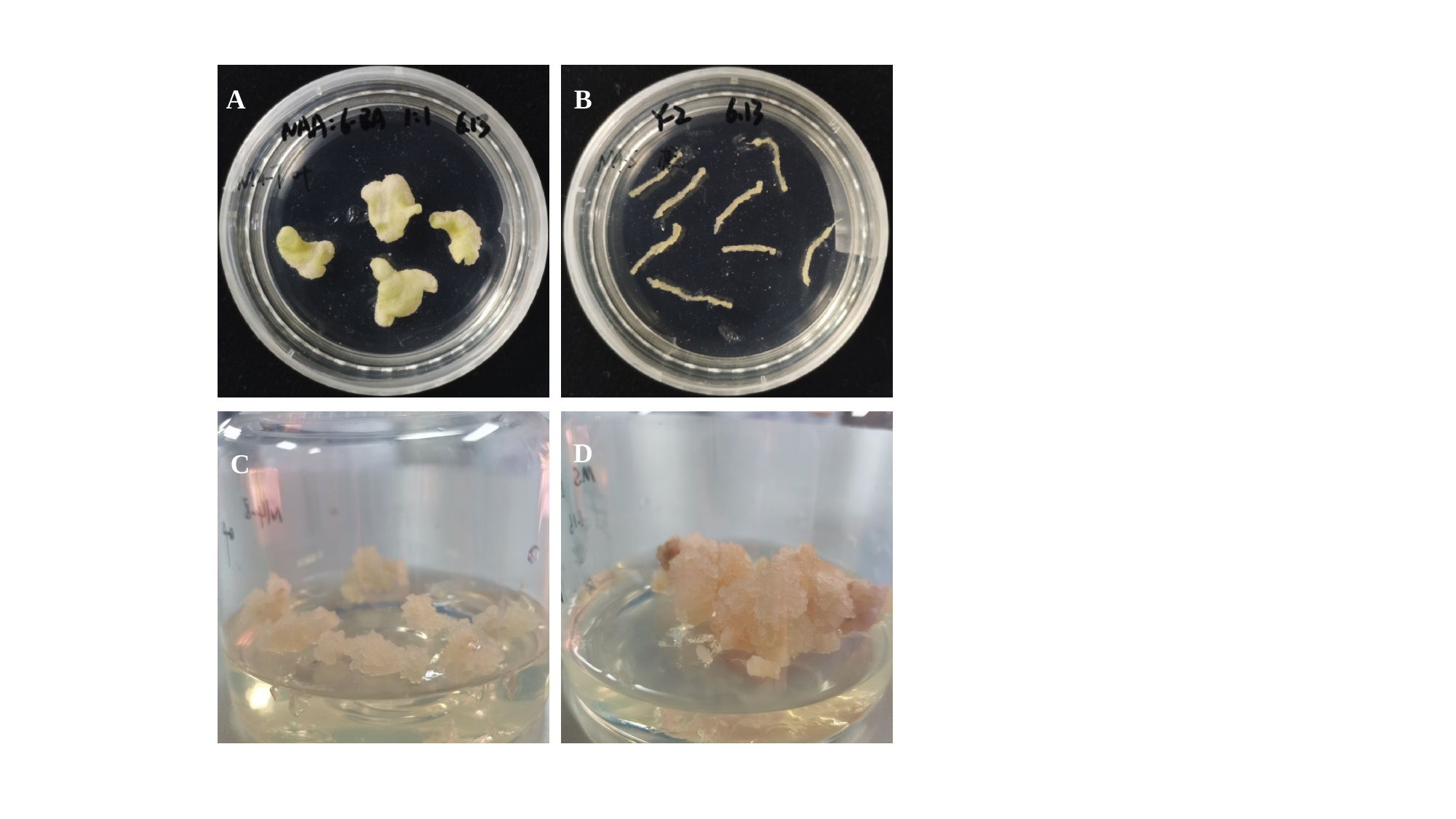

A
B
D
C

## Slide 2
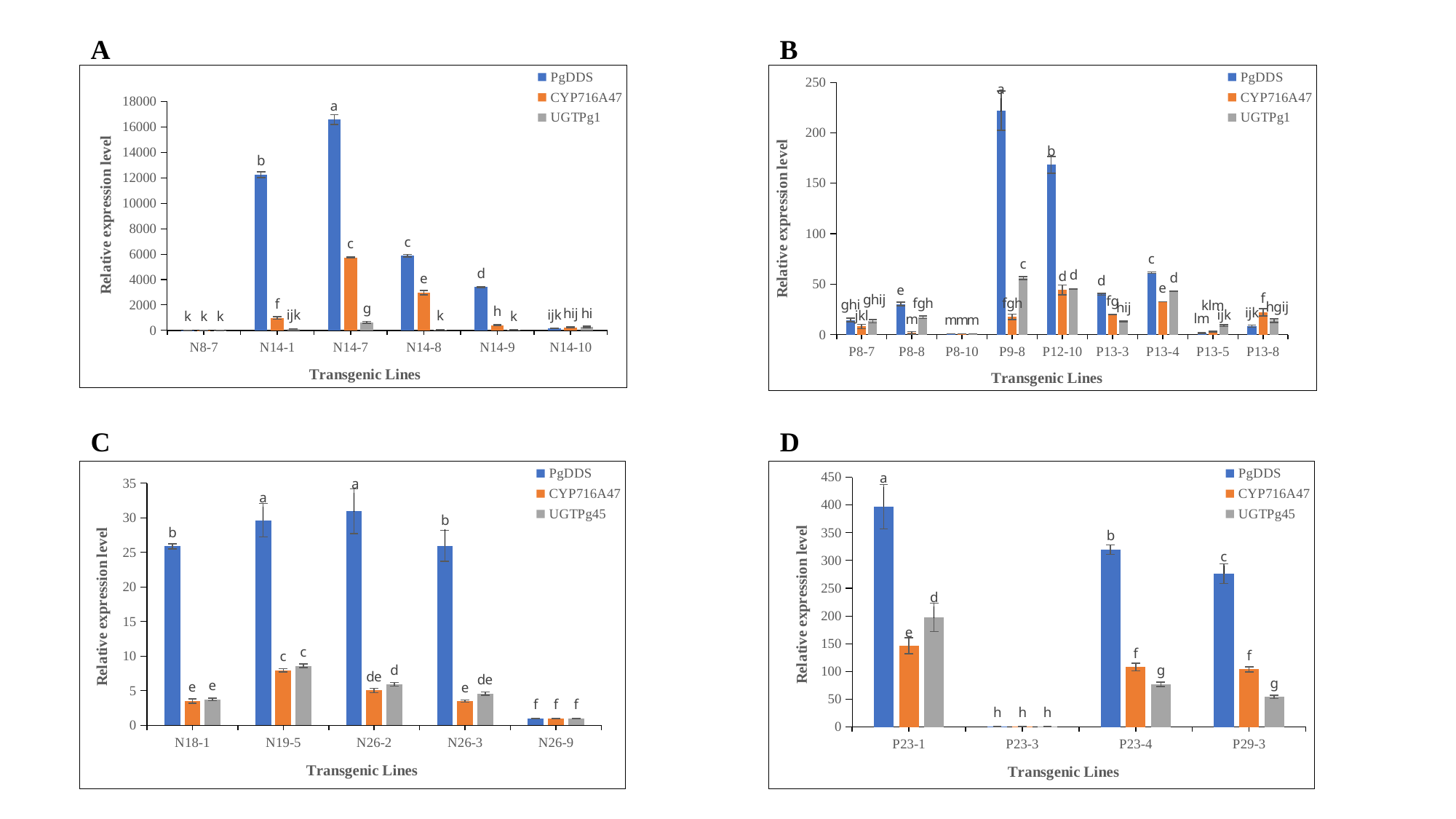

A
B
### Chart
| Category | PgDDS | CYP716A47 | UGTPg1 |
|---|---|---|---|
| N8-7 | 1.0 | 1.0 | 1.0 |
| N14-1 | 12260.995613333333 | 987.3804266666666 | 111.74293 |
| N14-7 | 16587.346256666668 | 5736.507136666667 | 632.41162 |
| N14-8 | 5869.080143333333 | 2975.6576999999997 | 63.69948 |
| N14-9 | 3411.3732233333335 | 416.04664333333335 | 35.514289999999995 |
| N14-10 | 143.77500333333333 | 243.89221333333333 | 269.2120733333333 |
### Chart
| Category | PgDDS | CYP716A47 | UGTPg1 |
|---|---|---|---|
| P8-7 | 14.476996666666667 | 8.11455 | 13.19656 |
| P8-8 | 30.321033333333332 | 1.7430866666666667 | 17.227256666666666 |
| P8-10 | 1.0 | 1.0 | 1.0 |
| P9-8 | 221.7782066666667 | 17.673076666666667 | 56.11426666666667 |
| P12-10 | 168.22991 | 44.17075333333333 | 45.354846666666674 |
| P13-3 | 39.95971 | 19.91344666666667 | 13.178873333333334 |
| P13-4 | 61.40946333333333 | 32.64492666666666 | 42.84793 |
| P13-5 | 1.8378666666666668 | 3.01229 | 8.9439 |
| P13-8 | 8.344213333333334 | 22.127956666666666 | 13.87876 |C
D
### Chart
| Category | PgDDS | CYP716A47 | UGTPg45 |
|---|---|---|---|
| N18-1 | 25.89265333333333 | 3.5224833333333336 | 3.74443 |
| N19-5 | 29.663876666666667 | 7.947900000000001 | 8.594926666666668 |
| N26-2 | 30.95078666666667 | 5.050016666666667 | 5.942699999999999 |
| N26-3 | 25.964506666666665 | 3.5066166666666665 | 4.589973333333333 |
| N26-9 | 1.0 | 1.0 | 1.0 |
### Chart
| Category | PgDDS | CYP716A47 | UGTPg45 |
|---|---|---|---|
| P23-1 | 396.9643533333333 | 146.30653666666663 | 197.35737666666668 |
| P23-3 | 1.0 | 1.0 | 1.0 |
| P23-4 | 319.54064666666665 | 107.94186333333334 | 76.78801333333334 |
| P29-3 | 276.4802466666667 | 103.93275666666666 | 54.39088999999999 |
